# Molecular Diversity Required for the Formation of Autocatalytic Sets

**DOI:** 10.3390/life9010023

**Published:** 2019-03-01

**Authors:** Wim Hordijk, Mike Steel, Stuart A. Kauffman

**Affiliations:** 1 SmartAnalytiX.com, Lausanne, Switzerland; 2 Biomathematics Research Centre, University of Canterbury, Christchurch 8140, New Zealand; mike.steel@canterbury.ac.nz; 3 Institute for Systems Biology, Seattle, WA 98109, USA; stukauffman@gmail.com

**Keywords:** autocatalytic sets, systems chemistry, random graphs, origin of life

## Abstract

Systems chemistry deals with the design and study of complex chemical systems. However, such systems are often difficult to investigate experimentally. We provide an example of how theoretical and simulation-based studies can provide useful insights into the properties and dynamics of complex chemical systems, in particular of autocatalytic sets. We investigate the issue of the required molecular diversity for autocatalytic sets to exist in random polymer libraries. Given a fixed probability that an arbitrary polymer catalyzes the formation of other polymers, we calculate this required molecular diversity theoretically for two particular models of chemical reaction systems, and then verify these calculations by computer simulations. We also argue that these results could be relevant to an origin of life scenario proposed recently by Damer and Deamer.

## 1. Introduction

Systems chemistry deals with the design and study of complex chemical systems, i.e., dynamic, self-organized, multi-component reaction networks. One example of such a complex chemical system is an autocatalytic set: a chemical reaction network in which all the molecules mutually catalyze each other’s formation from simpler building blocks. Such systems have been considered a crucial step in the origin of life [1,2,3], and have also been shown to exist in current metabolisms [4] and even entire ecosystems [5,6].

Autocatalytic sets have been experimentally constructed and studied in the laboratory [7,8,9,10,11], but these studies have so far been limited to relatively small examples. It is therefore useful to have a solid mathematical foundation that can be used to study the properties and dynamics of autocatalytic sets of arbitrary sizes. Such a mathematical foundation has been developed in the form of reflexively autocatalytic and food-generated (RAF; see below for a more detailed explanation) theory [12], which has also been applied successfully to study some of the existing experimental examples [13,14,15].

In the context of the origin of life, Damer and Deamer recently proposed that protocells originated not in deep sea vents, but in pools on land, subject to evaporation and refilling by rain or terrestrial sources such as streams [16,17]. They suggest that lipid vesicles underwent successive wet-dry cycles on the margins of such pools. Central to this scenario is the idea that the lipid vesicles each contain a vast library of peptide or RNA polymers. These undergo the “plastein reaction” [18]. During the wet part of the cycle, polymers cleave into smaller fragments. During the dry part of the cycle, the removal of water (a leaving group for peptide or RNA synthesis) drives the reaction to the right and larger polymers are formed. The net result of this is a random shuffling of the polymer library in each lipid vesicle and the population of these vesicles.

Damer and Deamer then speculate that this process will lead to vesicles with sets of polymers that can reproduce themselves. As these authors argue, “selection of vesicles encapsulating these polymers leads to stepwise increments toward the emergence of functional systems capable of growth, reproduction, and evolution” [16] (p. 873). In particular, they state: “An important aspect of the scenario is that the polymers are not constant, but instead are in a steady state system in which hydrolysis is balanced by synthesis. Therefore, the system is continuously experimenting, trending towards combinations of polymers that are more stable than other combinations, and polymers that have specific functions, the most important being those that can catalyze their own reproduction” [16] (p. 880).

This sounds very much like autocatalytic sets of polymers. However, one important question that can be asked is what the minimum size of such polymer libraries would need to be to have a high probability of an autocatalytic set to form. Since this is currently still impossible to investigate experimentally, theory and computer simulations are an indispensible tool to answer such questions. Here, we apply RAF theory to provide more insight into this issue.

This paper is organized as follows. In Section 2 we briefly review the notion of autocatalytic sets and its formalization, RAF theory. In Section 3 we then turn to two specific models of complex chemical reaction networks exhibiting the spontaneous emergence of autocatalytic sets of polymers; Kauffman’s binary polymer model and the Jain–Krishna model. In particular, we calculate theoretically the minimum diversity of polymers required for autocatalytic sets to emerge in each model, based on a given probability that an arbitrary polymer catalyzes an arbitrary reaction. We then verify these theoretical calculations with computer simulations and show that there is good agreement between the two. Finally, in Section 4 we review data that suggests the probability of catalysis to be on the order of $10^{-5}$ or higher, and discuss what this implies for the required molecular diversity for the existence of autocatalytic sets of polymers. We argue that it is quite plausible that this required diversity of polymers would have existed in lipid vesicles in the Damer and Deamer scenario, and that our results can thus provide theoretical support for such an origin of protocells.

## 2. Background: Autocatalytic Sets

The notion of autocatalytic sets was introduced by Kauffman [19,20,21]. Simply put, an autocatalytic set is a set of molecules that mutually catalyze each other’s formation through sequences of chemical reactions starting from a basic food source. More formally, an RAF set is a set R of reactions that satisfies the following two conditions:

1. Reflexively autocatalytic (RA): each reaction r∈R is catalyzed by at least one molecule type that is either a product of R or is present in the food set *F*; and
2. F-generated (F): all reactants involved in reactions in R can be created from the food set *F* by using a series of reactions only from R itself.

**Figure 1:**
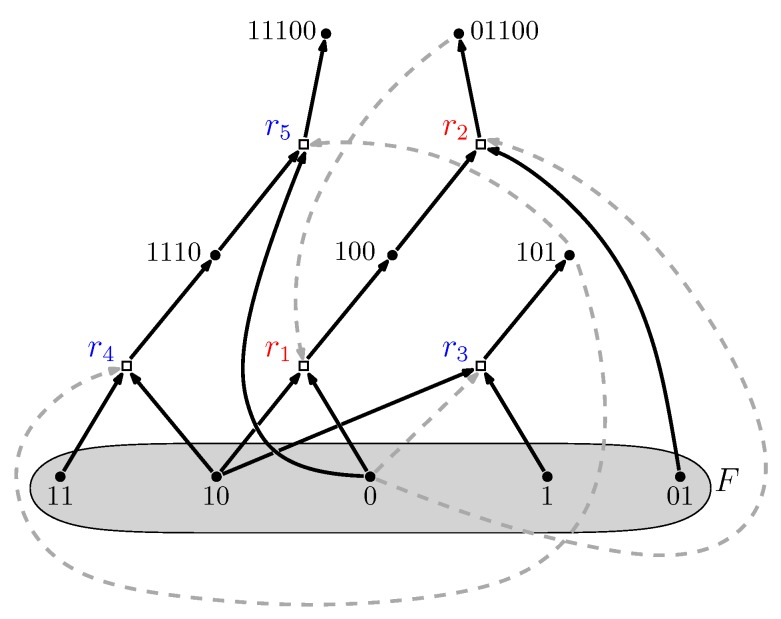
An example of a reflexively autocatalytic and food-generated (RAF) set that appeared in a simple binary polymer model where molecules are “bit string polymers” that can be ligated together into longer ones (see next section). Solid dots represent molecule types (labeled by bit strings); open squares represent reactions (ligations). Solid arrows indicate molecule types going into (reactants) and coming out of (products) a reaction; dashed grey arrows indicate which molecule types catalyze which reactions. The food set *F* consists of the monomers and dimers (i.e., bit strings of lengths one and two). Adapted from [24].

The food set *F* is a subset of molecule types that can be assumed to be available in the environment (i.e., they do not necessarily have to be produced by any of the reactions). A simple example of an autocatalytic (RAF) set is shown in Figure 1. A mathematically rigorous definition of RAF sets is provided in [22,23].

An autocatalytic set thus forms a catalytically closed (RA) and self-sustaining (F) reaction network (or RAF). In such a network, none of the individual molecules (polymers or other) need be self-replicators, but the set as a whole is capable of collective reproduction through mutual catalysis. Kauffman argued that “the achievement of the catalytic closure required for self-reproduction is an *emergent collective property in any sufficiently complex set of catalytic polymers*” (italics in original) [20] (p. 310).

Autocatalytic sets have been studied extensively both mathematically and computationally [12]. These studies have shown that RAF sets are highly likely to exist in simple polymer models, also at realistic (and modest) levels of catalysis (defined as the average number of reactions catalyzed per molecule type) [22,25,26]. Moreover, these results hold under a wide variety of model assumptions [23,27,28,29,30,31].

Furthermore, RAF sets often consist of many hierarchical levels of subRAFs [24,32]. For example, the RAF set in Figure 1, consisting of five reactions (labeled $r_{1}$ to $r_{5}$) contains the smaller subRAFs $\left\{r_{1},r_{2}\right\}$ (indicated in red) and $\left\{r_{3},r_{4},r_{5}\right\}$ (indicated in blue). This property provides one of the main necessary conditions for autocatalytic sets to be potentially evolvable [33,34,35,36].

However, autocatalytic sets are not just a theoretical concept. Several experimental examples have been created in the lab, either with nucleic acids or with proteins [7,8,9,10,11]. The earliest examples consisted simply of a system of two mutually catalytic nucleotide sequences, but later examples involved a set of nine peptides that mutually catalyze each other’s formation from shorter peptide fragments in various ways [9], or up to 16 ribozymes (catalytic RNA molecules) in a network of mutual catalysis [10]. Moreover, several of these experimental examples have been studied in more detail using the formal RAF framework, providing additional insights, and bringing theory and experiments closer together [13,14,15]. Finally, the RAF framework has also been applied to the metabolic network of *E. coli*, showing that it forms an autocatalytic set comprising almost the entire network [4].

## 3. Results: Required Molecular Diversity for RAF Sets

We now consider two particular models of chemical reaction systems that have been shown to exhibit autocatalytic sets. In both these models, catalysis is assigned randomly, according to some fixed probability. In other words, there is a given probability *p* that an arbitrary molecule type catalyzes any given reaction. For both models, we then calculate the required molecular diversity for autocatalytic sets to exist with high probability in (random) instances of these models, given a particular value of *p*.

### 3.1. Binary Polymer Model

In the binary polymer model, molecule types are represented by bit strings up to (and including) a maximum length *n*. The possible reactions are ligation and cleavage, i.e., combining two bit strings into a longer one or cutting a bit string into shorter pieces (keeping the maximum bit string length constraint into account). For each combination of a molecule type (bit string) and a reaction, it is decided with probability *p* whether that molecule type catalyzes that reaction. The food set consists of all monomers and dimers, i.e., bit strings of lengths one and two. This model was originally introduced by Kauffman to model polymer-like reaction networks [20,21]. Figure 1 shows an autocatalytic set that was found in an instance of this binary polymer model. These polymers could represent either proteins or nucleic acids (RNA), or a combination of both [30].

In this model, the number of molecule types is $\left|X\right|=2^{n+1}-2$ and the number of (bi-directional) reactions is $\left|R\right|=\left(n-2\right)2^{n+1}+4$ (from Equations (2) and (3) of [26]). The first of these equations follows from the identity $2+2^{2}+2^{3}+\cdots +2^{n}=2^{n+1}-2$ (noting that $2^{k}$ is the number of sequences of length *k*) while the second requires a more elaborate mathematical technique. In the following calculations we ignore the constants −2 and +4, respectively, as they play negligible roles, and ignoring them does not alter any of the results described below.

Following Kauffman’s original argument [20,21], consider a graph G=(V,E) where the node set *V* consists of the polymer types, i.e., all possible bit strings up to and including length *n*. Furthermore, if a bit string $v_{i}$ catalyzes a reaction that produces bit string $v_{j}$, then a (directed) edge $\left(v_{i},v_{j}\right)$ is included in the edge set *E*. Thus, the number of nodes in *G* is |V|=|X| and the expected number of edges in *G* is |E|=p|X||R|. We call *G* the catalysis graph. Note that *G* may have more than one edge in *E* between two nodes ($v_{i}$ and $v_{j}$) if $v_{i}$ catalyses more than one reaction that generates $v_{j}$. Let us now consider abstract simple random graphs of the type introduced by Paul Erdős and Alfred Rényi (we call these E-R graphs). A celebrated mathematical result states that a phase transition occurs in the structure of E-R graphs—from a highly disconnected graph to one that consists of a connected ‘giant component’ that contains a large connected portion of the graph—when the ratio of edges to vertices in the graph increases past the fraction $\frac{1}{2}$ [37,38].

Thus, if we apply a rough heuristic argument, autocatalytic sets in the binary polymer model might be expected to appear when

(1) $$\frac{p|X||R|}{\left|X\right|}=p\left|R\right|=p\left(n-2\right)2^{n+1}>\frac{1}{2}.$$

This can be rewritten as follows:

$$\left(n-2\right)2^{n+1}>\frac{1}{2p}$$

Taking logarithms to the base 2, this inequality can be rewritten as follow:

(2) $$n+\log_{2}\left(n-2\right)>\log_{2}\left(1/p\right)-2.$$

**Figure 2:**
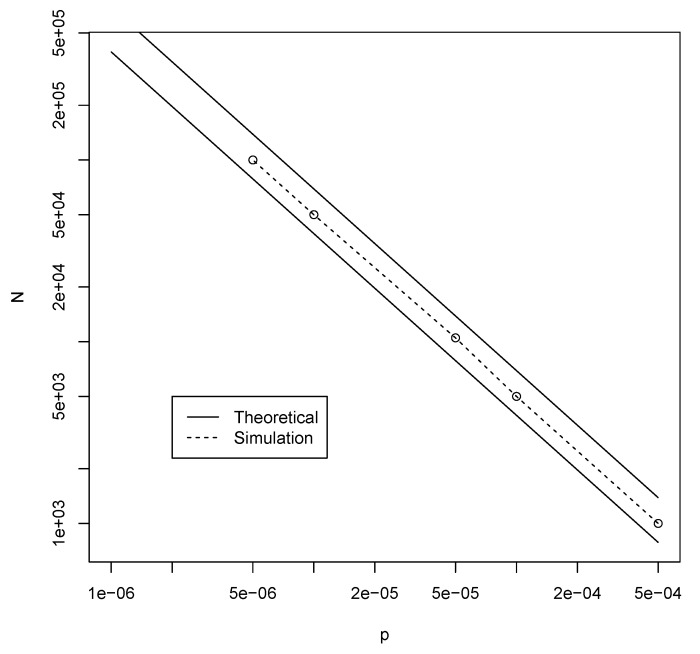
The theoretically calculated (solid line) and the simulation-based (dashed line) minimal values for *n* to get autocatalytic sets with high probability.

Thus, given a fixed probability of catalysis *p*, inequality (2) predicts what the minimum value for *n* (the maximum polymer length) is for which autocatalytic sets are likely to appear. For example, for $p\;=\;10^{-5}$, the right-hand side of inequality (2) becomes 14.61. Using n=11, the left-hand side gives a value of 14.17, which is just short of 14.61. However, n=12 gives a value of 15.32, which is larger than 14.61. So, for *n* at least 12, there should be a high probability that an instance of the binary polymer model with $p=10^{-5}$ will contain an autocatalytic set. Figure 2 shows the corresponding values for *n* (solid line) for various values of *p*, as derived from inequality (2).

**Figure 3:**
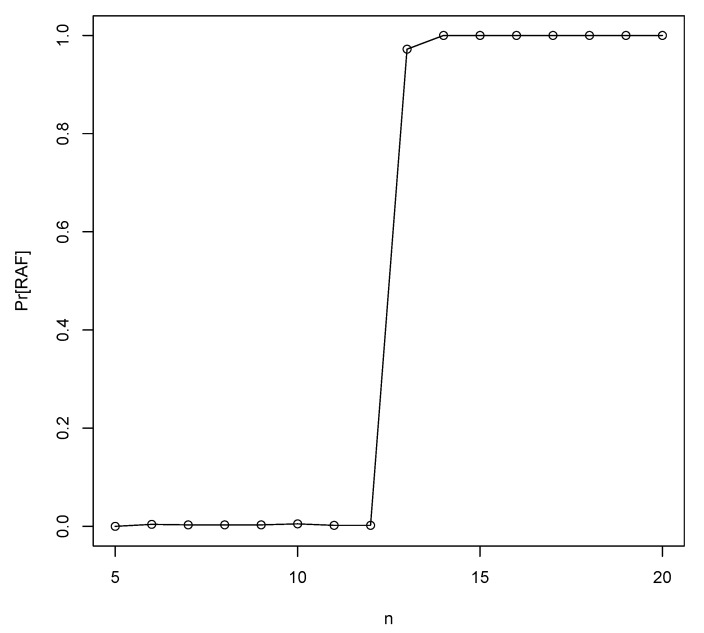
The probability of finding RAF sets (Pr[RAF]) for increasing values of the maximum polymer length *n* for a fixed probability of catalysis $p=10^{-5}$ in the binary polymer model.

Using the RAF algorithm [22] we can check whether these heuristically predicted values are correct, by creating many instances of the binary polymer model with various values of *p* and *n* and counting the fraction of instances that contain an autocatalytic (RAF) set. These simulation-based minimum values of *n* are also given in Figure 2 (dashed line). As the figure shows, the theoretical and simulated values agree very well. In fact, for each value of *p*, the simulation-based minimum value for *n* to get autocatalytic sets with high probability is larger than the theoretically calculated value by only 1. Figure 3 shows the simulated results for $p=10^{-5}$, clearly showing that n≥13 is sufficient to get autocatalytic sets with high probability.

It seems remarkable that the heuristically predicted and simulation-based numbers agree so well. In fact, despite Kauffman’s original argument, the Erdős and Rényi (E-R) results cannot be directly applied in this context for at least five reasons.

(1) The E-R result is specifically for undirected graphs, while the catalysis graph is a directed graph. Getting a connected component in an undirected graph is much easier than getting a (strongly) connected component in a directed graph.
(2) In the E-R setting the undirected graphs are simple (i.e., there is just a single edge between any two vertices) while in the setting described above there may be more than one edge.
(3) In the E-R setting there is an equal probability *p* that each pair of nodes has an (undirected) edge. In other words, E-R random graphs are isotropic, but the catalysis graph is non-isotropic.
(4) RAFs are required to be *F*-generated, and a giant connected component need not be (e.g., if the food set is a subset of the molecules not in the giant component).
(5) RAFs might form well before a giant connected component does (i.e., a RAF is not required, a priori, to be large).

Point (3) was noted by Kauffman already: “Their results do not directly apply to the connectivity properties of random catalyzed reaction subgraphs among peptide or RNA polymers, since those subgraphs are markedly nonisotropic, there being more reactions creating small polymers than reactions creating large polymers” [21] (p. 307).

Given this list of reasons for why E-R theory does not apply directly to the RAF setting, the question then arises: why are the predictions concerning the value of *n* at which RAFs appear relatively close to their exact values? We do not have a concise or complete explanation for this, but results from earlier work provide some clues. In [27] we showed that approximating a particular system (template-based catalysis) with a heuristically adjusted catalysis rate in a simpler (but incorrect) polymer model leads to very accurate predictions concerning RAF emergence. In that setting the model mis-specification was relatively minor—though arguably similar in spirit to point 3 above.

Furthermore, although it is not obvious, a mathematical result from [39] (Theorem 4) shows that point (5) plays a minor role, since there are no small RAFs at the level of catalysis at which RAFs first form in the binary polymer model (in contrast to a model we will describe in the next section). Point 2 is also likely to play a minor role at the level of catalysis at which RAFs are first forming. However the combination of these five reasons (and especially the first) give little confidence in any prediction.

Despite the success of the (mis-application of the) E-R heuristic in predicting the value of *n* for which RAFs appear, for larger values of *n* we expect the difference between the theoretical and simulation-based values to become larger. Nevertheless, this difference grows only very slowly (logarithmically) with increasing *n*. To see this, note that results from [26] (Theorem 4.1) imply that an instance of the binary polymer model will have a RAF (with probability, say, 95%) if:

(3) $$p\left(n-2\right)2^{n+1}=Cn\left(\mathrm{for}\;\mathrm{some}\;\mathrm{constant}\;C\right),$$

while in the E-R heuristic there is a constant *c* (e.g., c=0.5) in place of Cn. Now, taking logarithms (with base 2, as in inequality (2)), Equation (3) above becomes:

(4) $$\log_{2}\left(p\right)+\log_{2}\left(n-2\right)+\left(n+1\right)=\log_{2}\left(Cn\right)=\log_{2}\left(C\right)+\log_{2}\left(n\right),$$

while the E-R heuristic has only the term $\log_{2}\left(c\right)$ on the right-hand side. Since $\log_{2}\left(n\;-\;2\right)\;-\;\log_{2}\left(n\right)\;=\;\log_{2}\left(1-\frac{2}{n}\right)\;=\;o\left(1\right)$, the terms $\log_{2}\left(n-2\right)$ on the left-hand side and $\log_{2}\left(n\right)$ on the right-hand side of Equation (4) essentially cancel each other out for large enough *n*. In other words:

$$n=\log_{2}\left(C\right)-\log_{2}\left(p\right)-1+o\left(1\right),$$

while for the E-R case we have:

$$n^{*}=\log_{2}\left(c\right)-\log_{2}\left(p\right)-1-\log_{2}\left(n^{*}-2\right),$$

and so

$$n-n^{*}=\log_{2}\left(n^{*}-2\right)+O\left(1\right),$$

where O(1) refers the (asymptotic) constant $\log_{2}\left(C/c\right)+o\left(1\right)$. In other words, the difference between the minimally required value *n* (for the simulation-based analysis of the binary polymer model) and $n^{*}$ (for the E-R case) grows logarithmically with $n^{*}$. Thus, for values of *n* not too large, the E-R results provide a close enough theoretical estimation for the required maximum polymer length *n* to get RAF sets given a fixed probability of catalysis *p*.

**Figure 4:**
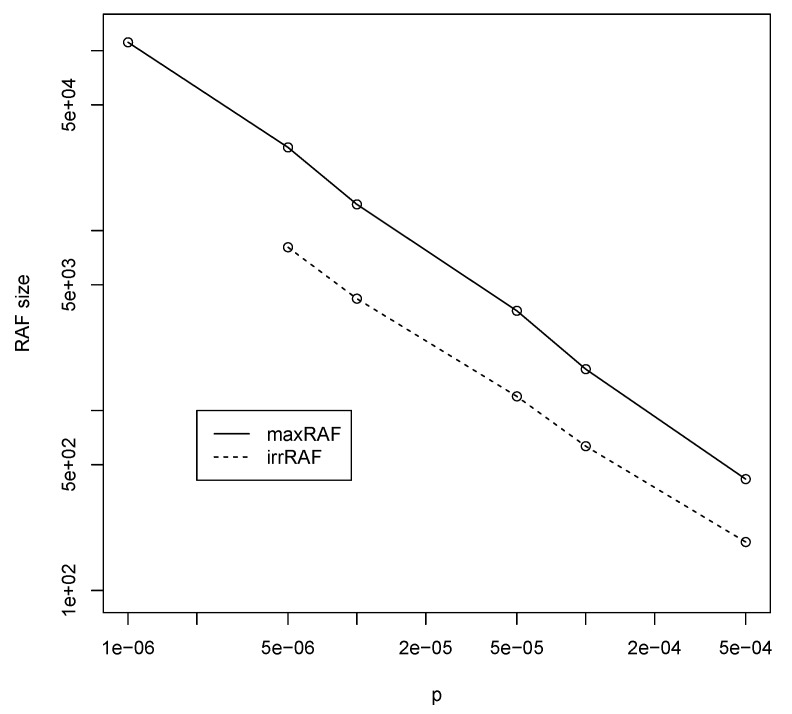
The average maxRAF (solid line) and irreducible RAF (irrRAF) (dashed line) sizes (in number of polymer types) for various values of *p* and corresponding minimally required *n* (as taken from Figure 2).

Finally, note that for $p=10^{-5}$ the maximum polymer length required for autocatalytic sets to form is n=13, which means the total number of polymer types is $\left|X\right|=2^{14}\approx 16,000$. However, from the simulation results using the RAF algorithm, it turns out that the average size of a (max)RAF set is about 14,000 polymer types. Furthermore, irreducible RAF sets (irrRAFs) are even smaller, just over 4000 polymer types on average. Figure 4 shows average maxRAF (solid line) and irrRAF (dashed line) sizes for different values of *p* and corresponding required *n*. These RAF sizes appear to follow a straight line in a log-log plot very closely, and thus decrease as a power law with increasing values of the probability of catalysis.

### 3.2. Jain–Krishna Model

**Figure 5:**
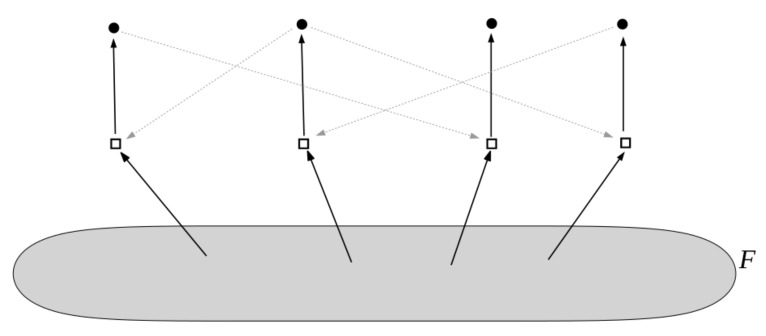
An example of an autocatalytic (RAF) set in the Jain–Krishna model. The four molecule types at the top of the reaction network are directly created from an implicitly assumed “generic” food set, and they mutually catalyze each others formation.

We now consider chemical reaction networks that have the property that every reaction has all its reactants in the food set, called “elementary” in [40]. Such networks arise in the Jain and Krishna model [41,42,43], where there are *N* molecule types (the vertices in the catalysis graph *G*), and there is a probability *p* that any molecule type $v_{i}$ catalyzes the formation of molecule type $v_{j}$, where each molecule type is directly produced from an implicitly assumed “generic” food set. An example of a RAF set in an instance of the Jain–Krishna model is presented in Figure 5. Note that in this model, the food-generated part in the RAF definition is automatically satisfied in any subset of reactions.

Thus, there are |V|=N vertices and $\left|E\right|=pN^{2}$ edges. To get a giant connected component, we again need $\left|E\right|/\left|V\right|=pN^{2}/N=pN>1/2$. For example, given a fixed probability of catalysis of $p=10^{-5}$, this gives $N>5\cdot 10^{4}$. However, a giant connected component is already much more than required to get an autocatalytic set in elementary reaction networks. In fact, any cycle (of any length) would constitute an RAF set, since we only need to check for the RA property. Thus, the study of the emergence of RAFs in elementary reaction networks is essentially equivalent to the study of the emergence of directed cycles in a random directed graph, a topic that was explored asymptotically in 1989 (motivated also by origin of life considerations) by [44]. Here we show that cycles already happen at much smaller values of *N* than in the binary polymer model, as stated in the following theorem.

**Theorem** **1.** *Consider a set S of N molecule types where each molecule type independently catalyzes, with fixed probability p, a given reaction that converts food molecule(s) to a molecule of S (we assume that no food molecule catalyzes any reaction). Let μ=Np be the expected number of molecules in S of which the formation is catalyzed by any given molecule in S. Then for all μ∈(0,1), the probability P that there is a nonempty subset $S^{\prime }$ of S for which every element of $S^{\prime }$ is produced by a catalyzed reaction satisfies the inequality*

1−exp(−μ)≤P≤−ln(1−μ).

**Proof.**  First, *P* is at least equal to the probability that at least one molecule $v_{i}$ from *S* catalyzes its own formation (since then we can take $S^{\prime }=\left\{v_{i}\right\}$). The probability of that event is $1-{\left(1-p\right)}^{N}$, which is greater or equal to 1−exp(−Np)=1−exp(−μ) (a similar lower bound can be derived for the probability of generating a cycle of length >1).

On the other hand, such a set $S^{\prime }$ having the property described in Theorem 1, exists if and only if there exists a directed cycle in the graph D=(S,A) where $\left(v_{i},v_{j}\right)$ is an arc (element of *A*) precisely if $v_{i}$ catalyzes a reactions that creates $v_{j}$. Now *P* is less or equal to the expected number of directed cycles in *D* (since for any non-negative integer-valued random variable *X*, one has P(X>0)≤E[X]). The expected number of directed cycles in *D* of length *k* is given by:

Nk·(k−1)!·pk=N(N−1)⋯(N−k+1)pkk,

since there are Nk ways to select *k* vertices from *S*, there are (k−1)! to arrange these *k* vertices into a directed cycle, and the term $p^{k}$ is the probability that each of the *k* arcs of the cycle is present. Thus,

$$E\left[X\right]=\sum_{k=1}^{N}N\left(N-1\right)\cdots \left(N-k+1\right)\frac{p^{k}}{k}\leq \sum_{k=1}^{\infty }\frac{{\left(Np\right)}^{k}}{k}=-\ln\left(1-\mu \right).$$

Thus P≤E[X]≤−ln(1−μ), as claimed. □

So, if we want to have autocatalytic sets (i.e., cycles) with probability at least, say, *P* = 0.50, then Theorem 1 gives us a lower and upper bound for the minimally required *N*:

1−exp(−μ)≤0.50≤−ln(1−μ)

Substituting μ=Np, after some straightforward algebra we get

(5) $$\frac{1-\exp(-0.50)}{p}\leq N\leq \frac{-\ln(0.50)}{p}$$

**Figure 6:**
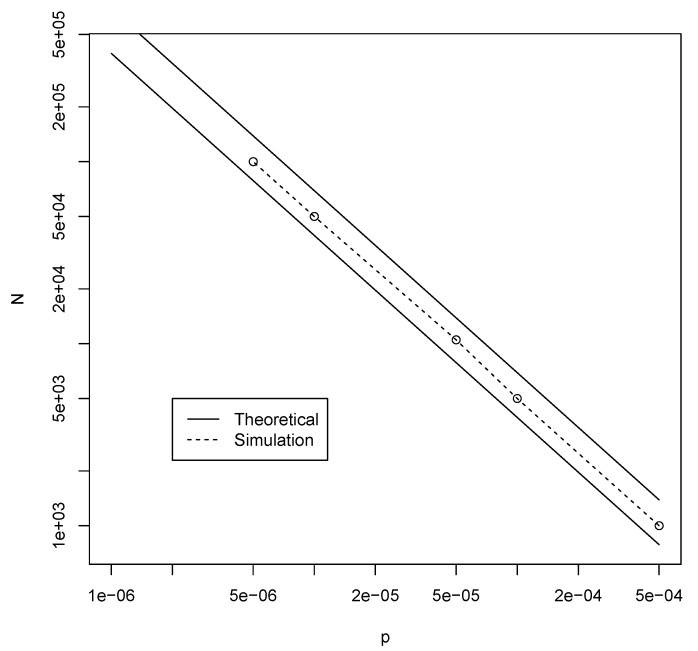
The theoretically calculated bounds (solid lines) and simulation-based values (dashed line) for the minimal value of *N* to get autocatalytic sets with probability at least P=0.50 in the elementary model.

Figure 6 shows these lower and upper bounds (solid lines) for the required values of *N* for the same values of *p* as in Figure 2 above. We can again use the RAF algorithm on many instances of the elementary model to check if these theoretically calculated values are correct. These simulation results are also shown in Figure 6 (dashed line), and indeed fall nicely within the theoretical bounds.

Note that for $p=10^{-5}$ about 50,000 molecule types are needed to get a RAF set. However, it turns out that these RAF sets consist on average of only about 4 molecule types. This is consistent with a result from [44] (Theorem 11), which shows that the length of the first cycles that form in a random directed graph has a (asymptotic) distribution that decays according to a power law with exponent −2. Thus we expect to see short cycles (of length one, two, and) with longer cycles becoming more rare at an inverse square rate.

The reason why many more molecule types are needed than in the binary polymer model is that in the elementary model, for each molecule type there is only one reaction that produces it. In other words, the ratio of reactions to molecule types is constant (in fact, it is exactly one), whereas in the binary polymer model this ratio grows as *n* (the length of the longest polymers). On the other hand, when autocatalytic sets do start to show up in the elementary model, they are very small, given that the Food-generated part of a RAF set is always trivially satisfied in any subset of molecule types in the elementary model.

The main results are summarized in Table 1, which presents the required polymer diversity to get RAF sets in both models for various values of the probability of catalysis *p*.

## 4. Discussion

In 1990, George Smith introduced “phage display” [45]. Here, short random DNA sequences are each cloned into a different bacteriophage into the gene that codes for a coat protein. This creates a library of millions of different phage, each of which, when mature, displays a different peptide on its coat. Smith carried out an experiment using monoclonal antibodies bound to a petri plate, and exposed the plate to $22\times 10^{6}$ phage. He then eluted phage which did not bind the antibody molecule on the plate, changed buffer and eluted phage which did bind, amplified the selected phage by infection into *E. coli*, and carried out a few successive rounds of selection and amplification. From the 22 million starting phage library, he selected 16 different phage encoding 16 different random peptides [45].

We conclude from this experiment that the probability a random peptide binds a random epitope is on the order of one in a million, or $10^{-6}$. This result depends partly on the binding affinity required in the elution step [45]. For a slightly milder elution procedure, a somewhat lower affinity would be sufficient, so the probability of binding would be higher. In fact, more recent experiments using phage display seem to indicate that the probability of a random peptide binding to the stable analogue of a transition state of a reaction is on the order of $10^{-4}$ [46] (Section 2.5). From an initial random library with a concentration of $10^{9}$ cfu/ml, a concentration of around $10^{5}$ cfu/ml was found to bind to the transition state analogue (TSA). This would imply a binding probability of $10^{5}/10^{9}=10^{-4}$, although this may be slightly too optimistic given that there was one round of elution and amplification first.

Lerner and others, in the decade of the 1990s, studied “catalytic antibodies” [47]. These researchers reasoned that if an antibody was able to bind to a chemically stable analogue of the transition state of a reaction, it would have a high probability to catalyze the reaction itself. Indeed, this is true. Monoclonal antibodies were selected that bound the TSA of a chosen reaction, and most of these monoclonal antibodies did, in fact, catalyze the reaction. We therefore conclude that the probably that a random peptide catalyzes a reaction is roughly equal to its probability of binding a random epitope or TSA.

Taking this probability of catalysis *p* to be on the order of $10^{-5}$, i.e., in between the more conservative estimate from [45] and the more optimistic estimate from [46], the results from Section 3.1 indicate that in the binary polymer model a maximum polymer length of n=13 is required for autocatalytic sets to arise with high probability. This implies a molecular diversity of around 16,000 polymer types (see also Table 1), with autocatalytic (RAF) sets for these parameter values containing on average 14,000 polymer types, while irrRAFs can be expected to contain just over 4000 polymer types.

For the Jain–Krishna model, the results from Section 3.2 indicate that the required molecular diversity would be at most 50,000, although the actual autocatalytic sets are very small, on average consisting of only four molecule types. In both the binary polymer model and the Jain–Krishna model, these number decrease rapidly for even higher probabilities of catalysis, as Figure 2 and Figure 6 and Table 1 show.

Bartel and Szostak examined the probabililyity of catalysis for RNA using a library of $1.6\times 10^{-15}$ random sequences and found 65 able to catalyze the desired reaction. Thus the probability of catalysis here is on the order of $10^{-13}$, i.e., orders of magnitude smaller than for peptides. This may be an underestimate, though. The probability of finding random RNA sequences that catalyze the reaction more slowly may be higher. However, this result does seem to suggest that peptides may have been more crucial in the formation of initial (prebiotic) RAFs than ribozymes.

On the other hand, there is increasing evidence that many organic reactions can be catalyzed by inorganic elements alone, such as metals and minerals [48,49,50,51]. In fact, many modern-day enzymes still use these inorganic elements as their cofactors [52,53]. So, it is possible that these inorganic elements could have helped “kick-start” the formation of an autocatalytic set, where the catalysis is then later on taken over by peptides and/or ribozymes, e.g., by incorporating the original inorganic catalysts as their cofactor. This way, more specific and more efficient catalysts can be produced by the system itself.

In that case, the “effective” probability of catalysis could be considered even higher, perhaps indeed on the order of $p=10^{-4}$. According to our results above, this means a maximum polymer length of only n=10 would suffice in the binary polymer model, i.e., a molecular diversity of around 2000 polymer types, with maxRAFs consisting (on average) of 1700 polymer types and irrRAFs of just over 600. In the Jain-Krishna model, a molecular diversity of around 5000 would suffice.

It is quite plausible that such diversities of polymers (2000 for $p=10^{-4}$ or even 16,000 for $p=10^{-5}$) would have been found in the lipid vesicles undergoing the plastein reactions in the Damer and Deamer scenario, randomly shuffling the polymer libraries in each vesicle on each wet dry cycle. Even more so since peptides have more than just two building blocks, even if only half of the current 20 amino acids would have been available at first. As Wieczorek et al. conclude: “Peptides whose length is between 10 and 40 residues (and more) can be formed, at least in principle, by spontaneous polymerization of activated amino acids followed by fragment condensation, with the help of short peptide catalysts that are themselves components of the peptide pool” [54] (p. 18). Thus, the spontaneous emergence of collectively autocatalytic sets of polymers can be expected given a constant turn-over of polymer diversity, which is basically the equivalent of producing ever new instances of, e.g., the binary polymer model. We therefore believe our results can provide strong theoretical support for the original Damer and Deamer scenario for the origin of protocells.

In conclusion, theoretical and simulation-based tools are useful in gaining more insight into questions that are still difficult to investigate experimentally, such as the required molecular diversity for the existence of autocatalytic sets in random polymer libraries. Systems chemistry deals with such complex chemical reaction networks by definition, and can thus clearly benefit from a solid mathematical foundation as well. In this paper, we have provided a specific example of such a theoretical contribution.

## Figures and Tables

**Table 1:** The required polymer diversity for the existence of autocatalytic sets for various values of the probability of catalysis *p*, for both the binary polymer model (BPM) and the Jain–Krishna model (JKM).

*p*	$10^{-6}$	$5\times 10^{-6}$	$10^{-5}$	$5\times 10^{-5}$	$10^{-4}$	$5\times 10^{-4}$
BPM	131,070	32,766	16,382	4094	2046	510
JKM	500,000	100,000	50,000	10,000	5000	1000

## References

[1] Kauffman S.A. (2007). Question 1: Origin of life and the living state. Orig. Life Evolut. Biosph..

[2] Hordijk W., Hein J., Steel M. (2010). Autocatalytic sets and the origin of life. Entropy.

[3] Nghe P., Hordijk W., Kauffman S.A., Walker S.I., Schmidt F.J., Kemble H., Yeates J.A.M., Lehman N. (2015). Prebiotic network evolution: Six key parameters. Mol. BioSyst..

[4] Sousa F.L., Hordijk W., Steel M., Martin W.F. (2015). Autocatalytic sets in *E. coli* metabolism. J. Syst. Chem..

[5] Cazzolla Gatti R., Hordijk W., Kauffman S. (2017). Biodiversity is autocatalytic. Ecol. Model..

[6] Cazzolla Gatti R., Fath B., Hordijk W., Kauffman S., Ulanowicz R. (2018). Niche emergence as an autocatalytic process in the evolution of ecosystems. J. Theor. Biol..

[7] Sievers D., von Kiedrowski G. (1994). Self-replication of complementary nucleotide-based oligomers. Nature.

[8] Kim D.E., Joyce G.F. (2004). Cross-catalytic replication of an RNA ligase ribozyme. Chem. Biol..

[9] Ashkenasy G., Jegasia R., Yadav M., Ghadiri M.R. (2004). Design of a directed molecular network. PNAS.

[10] Vaidya N., Manapat M.L., Chen I.A., Xulvi-Brunet R., Hayden E.J., Lehman N. (2012). Spontaneous network formation among cooperative RNA replicators. Nature.

[11] Arsène S., Ameta S., Lehman N., Griffiths A.D., Nghe P. (2018). Coupled catabolism and anabolism in autocatalytic RNA sets. Nucleic Acids Res..

[12] Hordijk W., Steel M. (2017). Chasing the tail: The emergence of autocatalytic networks. BioSystems.

[13] Hordijk W., Steel M. (2013). A formal model of autocatalytic sets emerging in an RNA replicator system. J. Syst. Chem..

[14] Hordijk W., Vaidya N., Lehman N. (2014). Serial transfer can aid the evolution of autocatalytic sets. J. Syst. Chem..

[15] Hordijk W., Shichor S., Ashkenasy G. (2018). The influence of modularity, seeding, and product inhibition on peptide autocatalytic network dynamics. ChemPhysChem.

[16] Damer B., Deamer D. (2015). Coupled phases and combinatorial selection in fluctuating hydrothermal pools: A scenario to guide experimental approaches to the origin of cellular life. Life.

[17] Deamer D.W. (2019). Assembling Life: How Can Life Begin on Earth and Other Habitable Planets?.

[18] Watanabe M., Arai S. (1992). The plastein reaction: Fundamentals and applications. Biochemistry of Food Proteins.

[19] Kauffman S.A. (1971). Cellular homeostasis, epigenesis and replication in randomly aggregated macromolecular systems. J. Cybern..

[20] Kauffman S.A. (1986). Autocatalytic sets of proteins. J. Theor. Biol..

[21] Kauffman S.A. (1993). The Origins of Order.

[22] Hordijk W., Steel M. (2004). Detecting autocatalytic, self-sustaining sets in chemical reaction systems. J. Theor. Biol..

[23] Hordijk W., Kauffman S.A., Steel M. (2011). Required levels of catalysis for emergence of autocatalytic sets in models of chemical reaction systems. Int. J. Mol. Sci..

[24] Hordijk W., Steel M., Kauffman S. (2012). The structure of autocatalytic sets: Evolvability, enablement, and emergence. Acta Biotheor..

[25] Steel M. (2000). The emergence of a self-catalysing structure in abstract origin-of-life models. Appl. Math. Lett..

[26] Mossel E., Steel M. (2005). Random biochemical networks: The probability of self-sustaining autocatalysis. J. Theor. Biol..

[27] Hordijk W., Steel M. (2012). Predicting template-based catalysis rates in a simple catalytic reaction model. J. Theor. Biol..

[28] Hordijk W., Hasenclever L., Gao J., Mincheva D., Hein J. (2014). An investigation into irreducible autocatalytic sets and power law distributed catalysis. Nat. Comput..

[29] Hordijk W., Wills P.R., Steel M. (2014). Autocatalytic sets and biological specificity. Bull. Math. Biol..

[30] Smith J., Steel M., Hordijk W. (2014). Autocatalytic sets in a partitioned biochemical network. J. Syst. Chem..

[31] Hordijk W., Steel M. (2016). Autocatalytic sets in polymer networks with variable catalysis distributions. J. Math. Chem..

[32] Hordijk W., Smith J.I., Steel M. (2015). Algorithms for detecting and analysing autocatalytic sets. Algorithms Mol. Biol..

[33] Vasas V., Fernando C., Santos M., Kauffman S., Sathmáry E. (2012). Evolution before genes. Biol. Direct.

[34] Hordijk W., Steel M. (2014). Conditions for evolvability of autocatalytic sets: A formal example and analysis. Orig. Life Evolut. Biosph..

[35] Hordijk W. (2016). Evolution of autocatalytic sets in computational models of chemical reaction networks. Orig. Life Evolut. Biosph..

[36] Hordijk W., Naylor J., Krasnogor N., Fellermann H. (2018). Population dynamics of autocatalytic sets in a compartmentalized spatial world. Life.

[37] Erdős P., Rényi A. (1959). On random graphs. Publ. Math..

[38] Erdős P., Rényi A. (1960). On the evolution of random graphs. Publ. Math. Inst. Hung. Acad. Sci..

[39] Steel M., Hordijk W., Smith J. (2013). Minimal autocatalytic networks. J. Theor. Biol..

[40] Steel M., Hordijk W., Xavier J.C. (2019). Autocatalytic networks in biology: Structural theory and algorithms. J. R. Soc. Interface.

[41] Jain S., Krishna S. (1998). Autocatalytic sets and the growth of complexity in an evolutionary model. Phys. Rev. Lett..

[42] Jain S., Krishna S. (2001). A model for the emergence of cooperation, interdependence, and structure in evolving networks. PNAS.

[43] Jain S., Krishna S. (2002). Large extinctions in an evolutionary model: The role of innovation and keystone species. PNAS.

[44] Bollobás B., Rasmussen S. (1989). First cycles in random directed graph processes. Discrete Math..

[45] Scott J.K., Smith G.P. (1990). Searching for peptide ligands with an epitope library. Science.

[46] Quintarelli A. (2011). Systems Optimization for the Selection of Phage Display Random Peptide Libraries. Ph.D. Thesis.

[47] Tramontano A., Janda K.D., Lerner R.A. (1986). Catalytic antibodies. Science.

[48] Schwartz A.W., de Graaf R.M. (1993). The prebiotic synthesis of carbohydrates: A reassessment. J. Mol. Evol..

[49] Zhang X.V., Martin S.T. (2006). Driving parts of Krebs cycle in reverse through mineral photochemistry. J. Am. Chem. Soc..

[50] Muchowska K.B., Varma S.J., Chevallot-Beroux E., Lethuillier-Karl L., Li G., Moran J. (2017). Metals promote sequences of the reverse Krebs cycle. Nat. Ecol. Evolut..

[51] Varma S.J., Muchowska K.B., Chatelain P., Moran J. (2018). Native iron reduces CO_2_ to intermediates and end-products of the acetyl-CoA pathway. Nat. Ecol. Evolut..

[52] Christen P., Mehta P.K. (2001). From cofactor to enzymes: The molecular evolution of pyridoxal-5’-phosphate-dependent enzymes. Chem. Rec..

[53] Rees D.C., Howard J.B. (2003). The interface between the biological and inorganic worlds: Iron-sulfur metalloclusters. Science.

[54] Wieczorek R., Adamala K., Gasperi T., Polticelli F., Stano P. (2017). Small and random peptides: An unexplored reservoir of potentially functional primitive organocatalysts. The case of Seryl-Histidine. Life.

